# Position in proton Bragg curve influences DNA damage complexity and survival in head and neck cancer cells

**DOI:** 10.1016/j.ctro.2024.100908

**Published:** 2025-01-03

**Authors:** Tim Heemskerk, Celebrity Groenendijk, Marta Rovituso, Ernst van der Wal, Wouter van Burik, Konstantinos Chatzipapas, Danny Lathouwers, Roland Kanaar, Jeremy M.C. Brown, Jeroen Essers

**Affiliations:** a Department of Molecular Genetics, Oncode Institute, Erasmus MC Cancer Institute, Erasmus University Medical Center, Rotterdam, the Netherlands; b Department of Radiation Science and Technology, Delft University of Technology, Delft, the Netherlands; c Research & Development, HollandPTC, Delft, the Netherlands; d University of Brest, INSERM, LaTIM, UMR1101, F-29200 Brest, France; e Optical Sciences Centre, Department of Physics and Astronomy, Swinburne University of Technology, Hawthorn, Australia; f Department of Vascular Surgery, Erasmus University Medical Center, Rotterdam, the Netherlands; g Department of Radiotherapy, Erasmus MC Cancer Institute, Erasmus University Medical Center, Rotterdam, the Netherlands

**Keywords:** Proton therapy, DNA Breaks, Double-stranded, Squamous cell carcinoma of head and neck, DNA damage, Linear energy transfer, Radiobiology, DNA repair, Cell survival, Tumor Suppressor p53-Binding Protein 1

## Abstract

**Background and purpose:**

Understanding the cellular and molecular effect of proton radiation, particularly the increased DNA damage complexity at the distal end of the Bragg curve, is current topic of investigation. This work aims to study *in vitro* clonogenic survival and DNA damage foci kinetics of a head and neck squamous cell carcinoma cell line at various positions along a double passively scattered Bragg curve. Complementary *in silico* studies are conducted to gain insights into the link between cell survival variations, experimentally yielded foci and the number and complexity of double strand breaks (DSBs).

**Materials and methods:**

Proton irradiations are performed at the HollandPTC R&D proton beamline, using a double passively scattered setup. A custom water phantom setup is employed to accurately position the samples within the Bragg curve. FaDu cells are irradiated at the proximal 36 % point of the Bragg peak, (P36), proximal 80 % point of the Bragg peak (P80) and distal 20 % point of the Bragg peak (D20), with dose-averaged mean lineal energies ($\overset{¯}{y_{D}}$) of 1.10 keV/μm, 1.80 keV/μm and 7.25 keV/μm, respectively.

**Results:**

Clonogenic survival correlates strongly with $\overset{¯}{y_{D}}$, showing similar survival for P36 (D_37%_=3.0 Gy) and P80 (D_37%_=2.9 Gy), but decreased survival for D20 (D_37%_ = 1.6 Gy). D20 irradiated samples exhibit increased 53BP1 foci shortly after irradiation, slower resolution of the foci, and larger residual 53BP1 foci after 24 h, indicating unrepaired complex breaks. These experimental observations are supported by the *in silico* study which demonstrates that irradiation at D20 leads to a 1.7-fold increase in complex DSBs with respect to the total number of strand breaks compared to P36 and P80.

**Conclusions:**

This combined approach provides valuable insights into the cellular and molecular effect of proton radiation, emphasizing the increased DNA damage complexity at the distal end of the Bragg curve, and has the potential to enhance the efficacy of proton therapy.

## Highlights

• Survival of FaDu cells correlates strongly with dose-averaged mean lineal energy.
• FaDu cells irradiated in distal end show more 53BP1 foci shortly after irradiation.
• Resolution of 53BP1 foci is slower after distal end irradiation.
• Large residual 53BP1 foci remain, 24 h after distal end irradiation.
• In-silico results show irradiation in D20 leads to increase in complex DSBs.

## Introduction

Over the past decades, interest in proton radiotherapy for tumor treatment has significantly increased. The central rationale for proton radiotherapy lies in its superior spatial dose distribution in tissue compared to conventional radiotherapy. Photons deposit the maximum dose right after tissue entrance, with the dose gradually declining thereafter. In contrast, protons deposit a relatively low dose at the entrance, with a sharp rise in dose towards the end of their range, forming a so-called Bragg peak [1]. This characteristic enables the delivery of the maximum dose directly to the tumor while sparing the surrounding healthy tissue, making it particularly beneficial for treating head and neck cancers due to the proximity of critical organs [2]. In addition to protons, heavy ions, such as helium (He) and carbon (C) ions, also exhibit similar Bragg peak characteristics, which contribute to their effectiveness in particle therapy. While proton therapy is widely used, one of its key advantages is the relatively lower cost of constructing proton therapy facilities compared to those required for other types of particle therapy, such as heavy ion therapy. This makes proton therapy a more accessible option for many healthcare systems.

Proton radiotherapy induces tumor cell death by causing DNA damage. Recent radiobiological research has increasingly focused on understanding the complex relationship between proton irradiation and DNA damage. As the understanding of DNA damage complexity deepens, it becomes crucial to elucidate the structural yields of these damages and their implications for cellular survival. A critical aspect is the investigation of DSBs, which are among the most lethal forms of DNA damage, particularly when occurring as complex clustered DNA damage [3]. Complex clustered DNA damage involves a DSB along with multiple other lesions around the damaged site [4]. Studying these types of DSBs allows researchers to unravel the mechanism of DNA damage induction and its impact on cell survival.

To further understand the distribution of DSBs, *in silico* studies can offer valuable insights into the types and complexities of DNA damage. This requires a thorough understanding of the proton energy deposition pattern at the cellular level and the accompanying Linear Energy Transfer (LET) at the specific location of the irradiated tissue or cell. Since proton energy and energy spread vary along the Bragg curve, the energy deposition pattern at the cellular level also changes, affecting DNA damage induction and resulting DNA damage structures. Variations in the complexity and distribution of DSBs along the Bragg peak may lead to differences in cell survival fractions at different positions.

Holland Proton Therapy Center (HollandPTC), one of three proton therapy facilities in the Netherlands, consists of a unique clinical Research & Development (R&D) proton beamline. This beamline consists of a double passively scattered setup, specifically designed to conduct radiobiological experiments for studying cellular responses to proton therapy and optimizing treatment efficacy. This work aims to study *in vitro* clonogenic survival and DNA damage foci kinetics of a head and neck squamous cell carcinoma cell line along the double passively scattered proton Bragg curve of HollandPTC. Complementary *in silico* studies – which involve computational simulations of proton energy deposition, DNA damage induction, and the resulting damage structure at the cellular level – will provide insights into the link between experimentally yielded foci and the number and complexity of DSBs. These computational models will help predict how different energy deposition patterns influence DNA damage and repair processes, enhancing the interpretation of experimental data. Understanding these interrelations and correlations is crucial for elucidating the complex mechanisms underlying proton-induced cellular responses. This integrated approach provides valuable insights into the effects of proton radiation at cellular and molecular levels, potentially enhancing the efficacy of proton therapy in cancer treatment.

## Methods

### Overview of experimental setup at HollandPTC

**Fig. 1:**
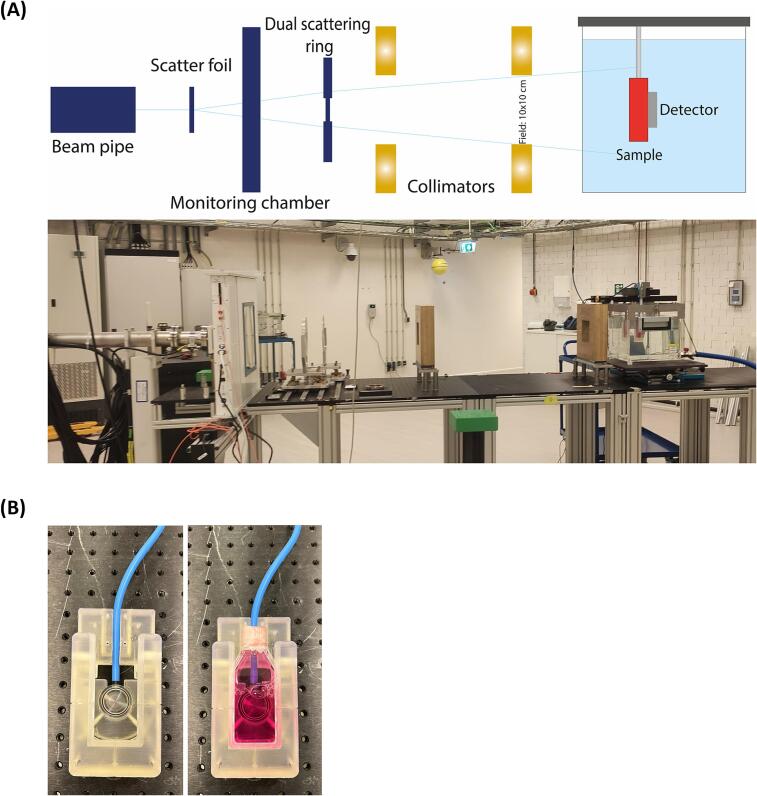
**Custom water bath setup to allow precise dosimetry and positioning of the sample.** (A) Schematic representation (top) and picture (bottom) of the R&D beam line setup at HollandPTC. The water bath combined with a linear motor allows for submillimeter precision in positioning the sample. (B) Photo of the sample holder. An Advanced Markus chamber is placed directly behind the T25 flask.

The HollandPTC R&D proton beamline was designed in a double passively scattered configuration optimized for a 150 MeV proton beam. The setup included a scattering foil, dual ring, and two-stage collimation system to enlarge and shape the initial pencil beam and obtain a homogeneous dose distribution across various field sizes. Fig. 1a depicts a photograph of the experimental bunker and a schematic representation of the setup, highlighting the beam line elements responsible for generating a uniform 10 × 10 cm^2^ field at the irradiation stage. Detailed descriptions of the experimental characterization of various radiobiological samples positioned within this setup are available in Rovituso et al [5].

In this study, a water phantom (30 × 30 × 30 cm^3^) equipped with a custom-designed 3D-printed holder attached to two motorized linear stages was used. This configuration allowed for the accommodation of a T25 cell culture flask and an Advanced Markus ionization chamber (type 34045, PTW Freiburg, Germany), positioned directly behind the flask as shown in Fig. 1b. Motorized linear stages enabled precise alignment of the T25 cell culture flask at the center of the 10 × 10 cm^2^ field, and allowed submillimeter-precision movement through the water phantom along the passively scattered Bragg curve.

For irradiation, three distinct positions along the passively scattered Bragg curve were selected, at the plateau, proximal 36 % point of the Bragg peak, (P36), proximal 80 % point of the Bragg peak (P80) and distal 20 % point of the Bragg peak (D20). To ensure accuracy and reproducibility, measurements were conducted prior to irradiation using the Markus chamber and a cell flask filled with cell medium to validate the precise location of the Bragg peak. Subsequently, the positions P36, P80, and D20 were determined, taking into account the plastic wall of the flask and the lid of the Markus chamber, ensuring precise irradiation of the flask at the specified positions. Throughout the irradiation process, the Markus chamber monitored the dose behind the T25 flask, ensuring adherence to the prescribed dose and precise positioning relative to the Bragg curve. Each flask was irradiated separately and the irradiation times were adjusted to account for the differences in dose rate at each position.

### Cell culture

FaDu cells (HTB-43, ATCC), derived from head and neck squamous cell carcinoma, were cultured in a 1:1 mixture of DMEM (4.5 g/L Glucose, with Ultraglutamine 1) and Ham’s F-10, supplemented with 10 % fetal calf serum and 1 % penicillin/streptomycin. Cells were maintained in a humidified incubator at 37 °C with 5 % CO_2_.

### Clonogenic survival assay

One day prior to irradiation, 1.5 × 10^6^ FaDu cells were seeded in T25 flasks and were allowed to attach overnight. Cells were irradiated with physical doses of 0, 2, 4 and 6 Gy at the three specified positions in the Bragg curve, with a dose rate of approximately 1.5 Gy per minute. Following irradiation, cells were trypsinized, counted and seeded in triplicates in 6 cm dishes. The number of cells seeded depended on the dose administered, with 300, 600, 1200, and 2400 cells per dish for the doses 0, 2, 4, 6 Gy, respectively. Colonies were allowed to form for 14 days, fixed and stained in Coomassie Blue staining solution (50 % methanol, 7 % acetic acid, 43 % demi water, 0.1 % brilliant blue R). Colonies were counted using the GelCount colony counter (Oxford optronic). To assess the relative sensitivity of cell survival curves, the data from three independent experiments were pooled and a linear-quadratic survival curve was fitted to the data points, using weighted least squares regression in GraphPad Prism 9. From the fitted curves, the dose needed to induce 37 % (D_37%_ (Gy)) and 10 % (D_10%_ (Gy)) survival was interpolated. Both 37 % and 10 % survival fall within our datapoints range allowing for interpolation from the curve.

### 53BP1 immunofluorescent staining

One day prior to irradiation, cells were seeded on 18 mm coverslips in 6-well plates at a density of 500.000 cells per well and allowed to attach overnight. Coverslips were transferred to a 12-well plate. The coverslips were attached to the well using PNIPAAm-PEG 3D thermoreversible hydrogel in cell culture medium (MBG-PMW20-1001, Mebiol Gel). The wells were filled with medium and sealed with Microseal B seals (MSB1001, Bio-Rad). The samples were irradiated with 2 Gy and fixed at time points 15 min, 2, 6, 8, 15, 18 and 24 h post-irradiation. Unirradiated coverslips were fixed after 15 min and 24 h. Cells were washed with phosphate buffered saline (PBS), fixed with 4 % paraformaldehyde in PBS for 15 min at room temperature and washed again with PBS. Cells were permeabilized with 0.1 % Triton X-100 in PBS for 2 × 10 min and blocked with PBS+ buffer (5 mg Bovine Serum Albumin and 1.5 mg glycine/mL PBS). Primary anti-53BP1 antibody (rabbit, 1:1000, NB100-304, Novus biologicals) was diluted in PBS+ buffer and cells were incubated with primary antibodies overnight at 4⁰C. After washing with PBS+ buffer, secondary antibodies (anti-rabbit Alexa488; Life Technologies) diluted in PBS+ buffer (1:1000) were applied, and cells were incubated in the dark for 1 h at room temperature. Coverslips were mounted on microscope slides using Antifade mounting medium with DAPI (Vectashield) and sealed with nail polish.

### Microscopy

Immunofluorescence in cells was visualized with a Leica STELLARIS 5 confocal microscope with the laser lines DAPI (405 nm) and Alexa 488 (488 nm). For each sample, four Z-stack images were captured using a 40x objective. Z-projections were generated and nuclear area, mean and integrated density of the DAPI signal were measured for each nucleus. Additionally, the number of 53BP1 foci, average focus size, and average focus intensity was analyzed for each nucleus using homemade ImageJ scripts. Cell nuclei were segmented based on the DAPI signal and nuclear area and integrated density of the DAPI signal were quantified using the measurement function within ImageJ. Foci within segmented nuclei were identified using thresholds based on the mean + factor * standard deviation of the 53BP1 signal [6]. The number, size, and intensity of segmented foci were measured using the measurement function within ImageJ.

To assess the resolution rate of 53BP1 foci, a sum of two exponential curves was fitted to the data points, using least squares regression in GraphPad Prism 9. The fitted curve follows the form:

$$\#Foci\;per\;nucleus\;=\;C_{1}\;\times \;e^{-\frac{t}{\tau_{1}}\;}-\;C_{2}\;\times \;e^{-\frac{t}{\tau_{2}}}\;+\;C_{3}\;\;\;\;\;\;\;C_{1},C_{2}>0.$$

Using this fitted curve, the half-life of 53BP1 resolution was determined.

### DNA damage complexity assessment using Geant4-DNA

**Supplementary Fig. 1:**
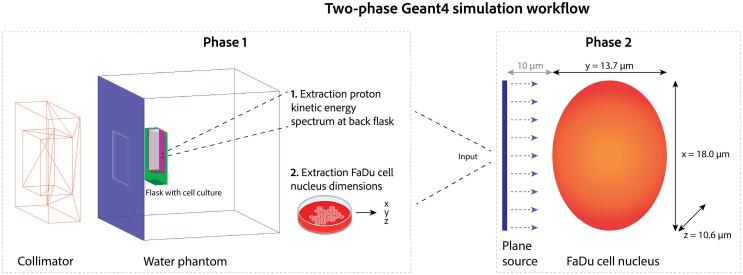
Two-phase Geant4 simulation workflow showing the first phase with the implemented experimental setup, where the estimated proton kinetic energy spectrum was extracted at the P36, P80 and D20. Simultaneously, the FaDu cell nucleus dimensions were extracted after staining and analysis in ImageJ. Both were used as input for the second phase Geant4 simulation, using a squared plane source 10 μm from the cell nucleus.

The estimated proton kinetic energy spectra at the back of the flask for the three specified positions along the passively scattered Bragg curve were obtained from Geant4 simulations [7], [8], [9], [19], [10], [11], [12], [13], based on the methodology developed by Groenendijk *et al.* [14], as illustrated in supplementary Fig. 1. Additionally, the FaDu cell nucleus dimensions in x, y and z were determined by fitting an ellipse onto the maximum projections of the microscopic z-projections, enabling extraction of the nucleus’ semi-axes. These proton kinetic energy spectra and FaDu cell nucleus dimensions were then used as input for the second phase (supplementary Fig. 1), which focused on studying DNA damage complexity along the 150 MeV passively scattered proton Bragg curve. Using the Geant4-DNA toolkit (Geant4 version 11.1.2), the “molecularDNA” example was employed to simulate the physical, physico-chemical and chemical stages of the particle interactions with biological media. With the fractalDNA tool, developed by Lampe *et al.* [15], [16], [17], a modified cell geometry was created to match the FaDu nucleus dimensions and the ∼ 6.4 Gbps of DNA content typical of normal human cells [18]. The Geant4-DNA option4 physics list [19] was adopted to simulate the physical and chemical interactions, including interactions of reactive species, which is the recommended physics list according to the Geant4-DNA collaboration [20]. The simulation parameters were used as presented in the work of Chatzipapas *et al.* [20], [21], subsequently simulating 2 × 10^4^ protons for each position in the Bragg curve (supplementary table 1).

The DNA damage quantification scheme, proposed by Nikjoo *et al*. [22], includes single strand breaks (SSB), DSBs, and their complexities (DSB+, and DSB++), as well as damage source types (direct, indirect, mixed, and hybrid). A DSB refers to lesions on opposite DNA strands within 10 base pairs. A more complex form, DSB+, includes a DSB accompanied by an additional lesion on one strand within 10 base pairs. An even more complex variant, DSB++, indicates the occurrence of at least two DSBs within a segment of the chromatin fiber, with a default segment length of 100 base pairs. These additional lesions can include a combination of various chemical alterations, such as oxidized bases, cross-links, or strand breaks [20]. Direct damage (DSB_D_) results from proton-DNA interactions, while indirect damage (DSB_I_) occurs as a result of interactions between protons and other cellular components, such as water, leading to the production of reactive oxygen species or other free radicals that cause strand damages. In cases where a DSB is attributed to both direct and indirect damage, it is classified as hybrid (DSB_HYB_), and if the DSB is caused by direct damage, together with another lesion caused by indirect damage, it is defined as DSB mixed (DSB_MIX_).

## Results

### Irradiation at D20 reduces clonogenic survival of FaDu cells

**Fig. 2:**
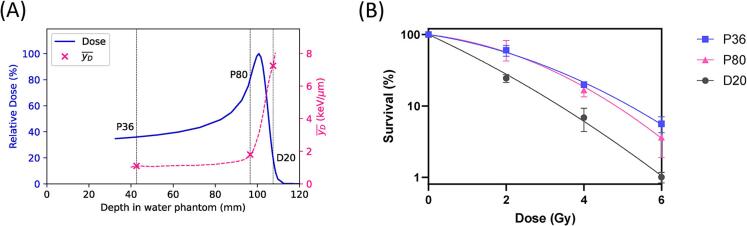
**Experimentally-informed simulated**$\overset{¯}{y_{D}}$**versus depth-dose distribution and clonogenic survival of FaDu cells through the Bragg curve.** (A) Depth vs relative dose distribution of the 150 MeV passively scattered proton Bragg peak in the water bath setup, along with the experimentally-informed simulated $\overset{¯}{y_{D}}$ values throughout the Bragg curve. The relative dose refers to the dose delivered at a given point along the proton Bragg curve, expressed relative to the maximum dose at the Bragg peak*.* Positions used for the biological experiments are indicated by the vertical dotted black lines. (B) Clonogenic survival of FaDu cells irradiated with proton radiation at the 3 positions in the Bragg curve indicated in Fig. 2. The mean survival of 3 replicate experiments was plotted. Error bars represent standard error of the mean.

The first aim of this study is to gain insights into the *in vitro* clonogenic survival of FaDu cells at different positions along the Bragg curve. A correlation is then sought between Bragg curve position associated FaDu cell survival and previously obtained experimentally-informed simulated $\overset{¯}{y_{D}}$ values at those positions [14]. By analyzing the clonogenic survival rates at these positions, it is intended to understand how variations in $\overset{¯}{y_{D}}$ affect cell survival. Fig. 2a shows the yielded experimentally-informed simulated $\overset{¯}{y_{D}}$ values in keV/μm along with the depth-dose distribution of the 150 MeV passively scattered proton Bragg curve, obtained from the work by Groenendijk *et al.* [14], where the relative dose refers to the dose delivered at a given point along the proton Bragg curve, expressed relative to the maximum dose at the Bragg peak. Fig. 2b presents the results of the clonogenic survival assay after irradiation of the samples at the P36, P80 and D20 positions. While the clonogenic survival of samples irradiated at P36 or P80 was not significantly different, a substantial reduction in the survival of FaDu cells was observed at the D20 position. At the distal end of the Bragg curve (D20), the dose required to achieve 37 % survival (D_37%_) was reduced by 1.88-fold compared to the plateau (P36) (Table 1). A slightly smaller decrease of 1.53-fold was observed for D_10%_. At this position, $\overset{¯}{y_{D}}$ is substantially higher (7.25 keV/μm) compared to P36 and P80 (1.10 and 1.80 keV/μm, respectively). The marked rise in $\overset{¯}{y_{D}}$ at D20 indicates more localized energy deposition by protons, leading to increased DNA damage and a significant decrease in cell survival.

**Table 1:** Overview of experimental and *in silico* results at the P36, P80 and D20, showing the D_37%_ and D_10%_ (Gy) determined from clonogenic survival, half-life of the 53BP1 foci (h), along with the experimental-informed simulated $\overset{¯}{y_{D}}$ values (keV/μm). The *in silico* results indicate the fraction of SSBs and DSBs with respect to the total amount of strand breaks, the SSB/DSB ratio and the fraction of complex DSBs (DSB + and DSB++) with respect to the total number of stand breaks.

**Position**	**Experimental**				***Insilico***			
	D_37%_ (Gy)	D_10%_ (Gy)	T_1/2_ 53BP1 foci (h)	$\overset{¯}{y_{D}}$ (keV/μm)	SSBs (#/Gy/Gbps)	DSBs (#/Gy/Gbps)	SSB/DSB	DSB+ & DSB++ / SB
P36	3.0	5.2	2.65 ± 0.36	1.10	291.66	9.51	29.7	0.48 %
P80	2.9	4.8	2.97 ± 0.01	1.80	282.91	10.02	27.2	0.55 %
D20	1.6	3.4	4.40 ± 0.17	7.25	260.54	11.62	21.4	0.88 %

### Altered 53BP1 foci kinetics after irradiation at D20

**Fig. 3:**
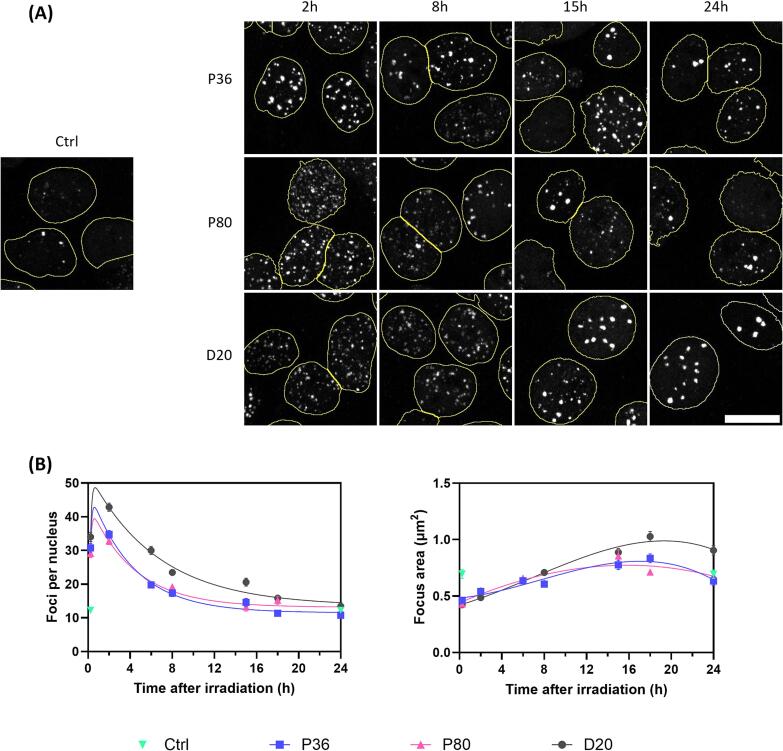
**53BP1 foci kinetics at different positions in the Bragg curve.** (A) Representative images of immunofluorescent 53BP1 staining of FaDu cells irradiated at the three indicated positions along the Bragg curve. Scalebar represents 15 μm. (B) Quantification of the 53BP1 foci kinetics over time. The number of 53BP1 foci per nucleus (left) and the average focus size per nucleus (right) are plotted over time. Error bars represent standard error of the mean. Experiment was performed 3 times, representative data of 1 replicate is shown here.

The second aim of this study is to further comprehend the observed FaDu cell survival outcomes through examination of the kinetics of the DSB repair protein 53BP1 at the three different positions in the Bragg curve. Analyzing the number and size of 53BP1 foci provides insights into the extent and complexity of DSBs at the specific positions. Representative images of the samples stained for 53BP1 after irradiation at the three different positions in the Bragg curve and fixed at different time points are shown in Fig. 3a. The general trend in the number of 53BP1 foci per nucleus is the same for all three positions, with a strong increase in foci after 15 min and 2 h, followed by gradual resolution of the 53BP1 foci (Fig. 3b). For the P36 and P80 samples, the kinetics of 53BP1 foci per nucleus are similar. At the D20 position, where $\overset{¯}{y_{D}}$ is significantly higher, the number of 53BP1 foci is significantly higher after two hours post-irradiation and resolves more slowly compared to the P36 and P80 positions. This delay in foci resolution suggests that the DSBs are more complex and more challenging to repair. In addition to the increased number of 53BP1 foci observed at D20, the size of these foci also shows notable differences. Directly after irradiation, the 53BP1 focus size decreases compared to the control sample. For the P36 and P80 positions, the foci size increases over time and aligns with the size observed in control samples after 24 h. This indicates a similar repair response to the induced DNA damage at these positions. Conversely, samples irradiated at D20 exhibit a sustained increase in foci size at 18 and 24 h post-irradiation. The larger foci suggest the presence of more clustered DSBs, which requires a more intensive repair response. To determine whether there is a difference in the rate of 53BP1 resolution, the sum of two exponential curves is fitted to the 53BP1 foci per nucleus (Fig. 3b). This analysis allows for the determination of the half-life of 53BP1 focus resolution at all three positions (Table 1). While the half-life is similar for the P36 and P80 samples (2.65 and 2.97 h respectively), the half-life of D20 irradiated samples is longer at 4.40 h. These findings indicate that shortly after irradiation, D20 samples exhibit a higher number of 53BP1 foci that are resolved more slowly. 24 h after irradiation at the D20 position, nearly all foci have been resolved, but the residual 53BP1 foci are larger than at the other two positions. The persistence and size of these foci, observed 24 h post-irradiation, suggest the presence of complex DNA damage.

### DNA damage complexity assessment using Geant4-DNA

**Fig. 4:**
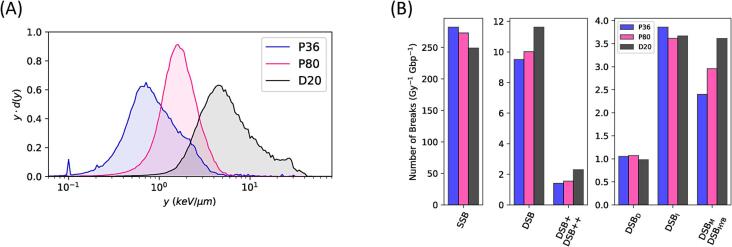
**Lineal energy spectra and DNA damage yields.** (A) The acquired experimentally-informed simulated lineal energy spectra at the P36, P80 and D20 of the Bragg curve. (B) The acquired DNA damage yields using the “molecularDNA” example based on break classification showing single strand breaks (SSBs), double strand breaks (DSBs) and the more complex DSBs: DSB + and DSB++. Additionally, DSBs based on source type are shown: DSBs as a result of direct damage (DSB_D_), DSBs as a result of indirect damage (DSB_I_) and hybrid or mixed DSBs. (DSB_M_ / DSB_HYB_).

To gain a deeper understanding and more comprehensive interpretation of the observed experimental results, Geant4-DNA simulations were used as a complementary tool to explore the complexity of DNA damage and provide additional insights into the biological processes. Fig. 4a illustrates the range of lineal energy values at each position along the Bragg curve, with $\overset{¯}{y_{D}}$ listed in Table 1. The corresponding proton kinetic energy spectra with mean energies of 88.2 MeV, 33.6 MeV and 12.9 MeV at P36, P80 and D20, respectively, are presented in Groenendijk *et al.* [14].

Comparison of DNA damage yields by complexity and source following proton irradiation using three distinct proton kinetic energy spectra reveals several key insights into the type and amounts of damage induced in the modeled FaDu cell nucleus. Analysis of break complexity, as depicted in Fig. 4b, demonstrates that while the number of SSBs decreases, the number of DSBs increases towards the distal end of the Bragg peak. Specifically, the contribution of DSBs to the total number of strand breaks is 3.3 % at P36 and 3.5 % at P80, rising to 4.5 % at D20. This results in a reduction of the SSB-to-DSB ratio from 29.7 and 27.2 at P36 and P80, respectively, to 21.4 at D20 (Table 1). Among the total DSBs induced, the fraction of complex DSBs (DSB + and DSB++) is 15 % at both P36 and P80 and rises to 20 % at D20. Relative to the total number of strand breaks, this represents a 1.7-fold increase in complex DSBs at the distal end of the Bragg peak compared to P36 and P80. Furthermore, analysis of the source of DSBs reveals that the primary contribution to double strand breakage comes from indirect damage, although no specific trend is observed along the Bragg curve. DSBs induced through a combination of direct damage and other reactions (hybrid or indirect damage) are mostly present at D20, supporting the observation of more complex DSBs at the distal end of the Bragg curve.

## Discussion

This study presents a novel approach by integrating experimental and *in silico* analyses to investigate the effects of proton radiation along the Bragg curve on cellular survival and DNA damage. While prior studies have focused primarily on single proton energies or broader LET ranges, our work uniquely combines: 1) Experimental data: detailed analysis of clonogenic survival and 53BP1 foci kinetics across three distinct positions (P36, P80, and D20) along the Bragg curve of a passively scattered 150 MeV proton beam. 2) Simulated DNA damage complexity: experimentally-informed simulations of DNA damage complexity using Geant4-DNA, which correlate mean lineal energy values with increased double-strand breaks (DSBs) and higher proportions of complex DSBs. 3) Integrated interpretation: directly linking experimental outcomes, such as reduced clonogenic survival and delayed 53BP1 foci resolution at D20, with simulation-derived insights into DNA damage complexity at this position.

This integrated approach not only elucidates the biological effects of high LET at the distal end of the Bragg curve but also provides mechanistic insights into the increased radiobiological effectiveness observed in proton therapy. By directly correlating LET, DNA damage complexity, and cellular responses, our work advances the understanding of proton radiation effects in a way that prior studies, limited to isolated experimental or simulation approaches, have not achieved.

These findings underscore the importance of integrating experimental data with simulations to improve the understanding of proton therapy's efficacy, potentially informing treatment optimization strategies for cancer patients.

The FaDu cell line was selected as a well-established model for head and neck squamous cell carcinoma (HNSCC), a major indication for proton therapy. Focusing on a single cell line enables us to analyze the relative effects of different positions along the Bragg curve without the confounding variability of inter-cell line differences, such as distinct radiation sensitivities and repair capacities.

A lower survival of FaDu cells is observed when samples are irradiated at the distal end of the Bragg curve, a result attributed to the higher $\overset{¯}{y_{D}}$ at this position, while the obtained D_37%_ and D_10%_ values for the P36 and P80 positions correlate well with other literature on X-ray and low LET proton radiation [23], [24], [25]. These results are consistent with previous research showing increased radiobiological effectiveness when LET increases. This has been demonstrated in various experimental models with protons of various energies, but also with heavier ions [26], [27], [28], [29], [30], [31]. The reduced survival of FaDu cells at D20 is accompanied by an increase in the number of 53BP1 foci and delayed foci resolution compared to P36 and P80. This observation is supported by the simulations indicating an increased contribution of DSBs at D20, which aligns with the observed experimental outcomes. In addition to the relatively increased DSBs at D20, the contribution of complex DSBs increases as well, indicating greater DNA damage complexity at the distal end of the Bragg peak. These complex breaks are more challenging to repair, leading to residual damage, which manifests as persistent 53BP1 foci with increased size. Such residual breaks can eventually lead to cell death, thereby decreasing cellular survival at this position.

The increased damage complexity with higher LET is thought to contribute to the observed increase in radiobiological effectiveness. Studies on plasmid DNA irradiated with various LET have demonstrated that higher LET leads to more fragmented and complex damage, as shown by atomic force microscopy [32], [33] and electrophoresis [33]. Our study focuses on 53BP1 foci kinetics, which reflect complex DNA damage associated with high-LET radiation. Higher LET radiation induces more complex DNA damage, which takes longer to repair. This is consistent with previous studies on DSB induction and repair using γH2AX or 53BP1 as DSB markers have demonstrated that higher LET results in increased break induction, followed by slower repair, with 53BP1 foci half-lives ranging from approximately 1 to 5 h after proton radiation [34], [35], [36], and more residual breaks after 24 h [37], [34], [38], [39]. Additionally, higher LET can lead to increased 53BP1 focus size or intensity [40], [41]. The results presented here are consistent with these findings, as they align with the observed increase in DNA damage complexity and residual breaks. This correlation is further supported by the *in silico* study, which also indicates elevated damage complexity at higher LET.

Previous research has utilized the Geant4-DNA application to investigate DNA damage yields induced by ionizing radiation in human fibroblasts cells, bacterial cells and human cells [16], [20], [21], [42], [43], [44]. However, studies examining proton-induced DNA damage have primarily focused on single proton energies. This approach complicates direct comparisons with the current work, which incorporates three distinct proton energy spectra. The complexity is particularly evident at the distal end of the Bragg peak, which consists of a broad spectrum of LET values (0.08–40 keV/µm [45]), making direct comparisons with a single LET value challenging. Furthermore, when considering the mean LET values corresponding to the three proton energy spectra used in this study, as shown in Table 1, the available LET range is relatively narrow. This contrasts with all studies that examine a broad range of proton energies and LET values (from a few keV/µm up to 60–80 keV/µm or more), which tend to observe more pronounced differences in their results. Despite the narrower LET range, this work is unique in its detailed investigation of proton-induced DNA damage along a proton Bragg curve. Nevertheless, assuming the corresponding mean LET values to the three proton energy spectra, several comparisons can be made with other studies. Sakata *et al*. evaluated DNA damage in a human cell nucleus model induced by protons [44], [46]. Their findings indicated a decrease in SSB yield as a function of LET, a trend also observed in this study. Additionally, an increase in DSB yield with increasing LET was observed, and the corresponding decreasing SSB/DSB ratio. Similar trends regarding SSB and DSB yields have been observed in other studies as well [21], [42]. Chatzipapas *et al*. (2022) simulated DNA damage using the “molecularDNA” example and analyzed DNA damage yield using the “human cell” example using mono-energetic proton energies varying between 0.15 and 66.5 MeV. They confirm that the particle interactions become denser with increasing LET, therefore exhibiting a higher probability for DSB occurrence [47]. This observation is reflected in the SSB/DSB ratio which indicates that SSBs form DSBs more often, reflected by a DSB:SB fraction of 3.3, 3.5 and 4.5 % at P36, P80 and D20, respectively.

Additionally, the majority of the works that study proton-induced DNA damage have been limited to analyzing only SSBs and DSBs and their corresponding ratios at different proton energies. This research, however, extends beyond to explore the more complex forms of DSBs. Because of this, comparisons between DNA damage complexity and the associated source of the breaks are difficult to make. There have been studies on the contribution of indirect damage with increasing LET which stated that indirect damage decreased as LET increased, but this was only studied at higher LET values (20 to 2106 keV/µm) [48]. The discrepancy seen in this work could be attributed to the lower LET range examined, or differences in the specific conditions and models used. Finally, as highlighted in several studies, it is important to note that the quantification of SSBs and DSBs and their complexity is highly sensitive to the damage scoring parameters [16], [21], [46].

The integration of DNA damage simulations with radiobiological experiments has proven highly valuable, as it provides deeper insights into how variation in radiation quality affect biological outcomes. This approach facilitates a direct correlation between LET, DNA damage complexity and survival of FaDu cells across different positions along the Bragg curve. Furthermore, the observed differences in 53BP1 foci kinetics and size correlate strongly with the increased DNA damage complexity identified in the simulations. Simulations alone cannot directly link to biological outcomes like cell survival or DNA damage repair kinetics, while experimental data provides only indirect measures of DNA damage complexity. Thus, the combination of both approaches enables a more comprehensive understanding of the relationship between radiation quality and its biological effects.

The clear correlation between DNA damage complexity and cell survival opens up several interesting questions. One critical question is how increased DNA damage complexity affects the corresponding repair mechanisms. Cells have several DSB repair pathways available, including non-homologous end joining (NHEJ), homologous recombination (HR) and theta-mediated end joining (TMEJ), each of which plays a role depending on the structure of the break [49]. Research has shown that irradiation with high LET particles leads to increased DNA break end resection in both G1 and S/G2 cells, suggesting a reliance on TMEJ and HR. However, significant differences in resection were achieved only with heavy-ion radiation, which has much higher LET compared to protons [41], [50], [51]. While some studies show a stronger dependence on HR for repairing proton-induced DNA damage compared to photon-induced DNA damage [52], [53], others suggest this dependence is specific for high LET protons [54], [55] or occurs primarily after carbon-ion irradiation [56]. Other groups have not consistently demonstrated a stronger dependence on HR after proton radiation [57], [58]. While loss of NHEJ typically results in significant radiosensitization, this effect appears to be mostly independent of the type of radiation used [52], [53], [54], [56], [57], [58], [59]. Thus, the dominant repair pathway under different circumstances remains unclear.

While our study primarily focuses on DNA double-strand breaks and complex strand breaks induced by proton radiation, we acknowledge the significant contribution of base damage through indirect action, such as hydroxyl radical-induced DNA lesions. These types of damage can lead to a variety of downstream biological effects, including cell aging and distant metastasis [60].

The presented combination of biological experiments with simulation data presents a promising approach to unravel the mechanisms behind pathway choice. By combining radiobiological experiments with cells deficient in specific repair pathways, together with simulations, it may be possible to construct a comprehensive framework to gain more insight into the underlying mechanism of pathway choice. This approach could offer valuable insights into the mechanism guiding pathway selection and contribute to optimizing proton therapy by enhancing the understanding of the cellular and molecular effects of proton radiation.

## Conclusion

This study demonstrates the relationship between DNA damage complexity and the survival of head and neck squamous cell carcinoma cells irradiated at various positions along the Bragg curve. *In vitro* clonogenic assays and 53BP1 foci kinetics were employed to evaluate cell survival and the number and size of 53BP1 foci. These experimental observations were complemented by *in silico* studies to elucidate the relationship between increased $\overset{¯}{y_{D}}$, corresponding decreased cell survival and DNA damage complexity. The results indicate that cells irradiated at the distal end of the Bragg curve (D20) exhibit lower survival rates, increased 53BP1 foci, delayed foci resolution, and larger foci sizes compared to the plateau position and right before the Bragg peak. These findings are attributed to the higher LET and increased complexity of DNA damage at D20, as supported by simulation data showing a higher proportion of DSBs and complex DSBs at this location. These complex forms of DNA damage are more challenging for cells to repair efficiently, leading to persistent damage and reduced cellular survival. In contrast, irradiating cells at the plateau and at the P80, where LET is lower, results in almost identical biological outcomes. This correlation between position in the Bragg curve, LET, and DNA damage complexity showed novel insights into the effects of proton radiation at cellular and molecular levels, which will be crucial to increase the efficacy of proton therapy in cancer treatment.

**Funding Statement**

This work was supported by Varian Medical Systems (a Siemens Healthineers Company) (PROTON-DDR, Grant No: 2019020 and Grant No: 2019011), the Dutch Cancer Society (INTOPROT, 12092/2018) and is part of the Oncode Institute, which was partly financed by the Dutch Cancer Society. The simulations were undertaken on the Dutch national e-infrastructure with the support of SURF Cooperative (Grant No: EINF-486 (2020)). This project is partly financed by the Surcharge for Top Consortia for Knowledge and Innovation (TKIs) from the Ministry of Economic Affairs and Climate. J. M. C. Brown was supported by a Veni fellowship from the Dutch Organization for Scientific Research (NWO Domain AES Veni 16808 (2018)) during the time of this study. K. P. Chatzipapas was partially funded from the European Union’s “EURATOM” research and innovation program (Piano Forte Partnership for Radiation Protection Research) under the 101061037 grant agreement (LutADose).

## CRediT authorship contribution statement

**Tim Heemskerk:** Conceptualization, Methodology, Investigation, Writing – original draft, Writing – review & editing. **Celebrity Groenendijk:** Conceptualization, Methodology, Investigation, Software, Writing – original draft, Writing – review & editing. **Marta Rovituso:** Methodology, Writing – review & editing. **Ernst van der Wal:** Methodology. **Wouter van Burik:** Investigation, Methodology. **Konstantinos Chatzipapas:** Software. **Danny Lathouwers:** Conceptualization, Supervision, Funding acquisition, Writing – review & editing. **Roland Kanaar:** Conceptualization, Supervision, Funding acquisition, Writing – review & editing. **Jeremy Brown:** Conceptualization, Supervision, Funding acquisition, Writing – review & editing. **Jeroen Essers:** Conceptualization, Supervision, Funding acquisition, Writing – review & editing.

## Declaration of competing interest

The authors declare that they have no known competing financial interests or personal relationships that could have appeared to influence the work reported in this paper.
