# Supplementary material for: Position in proton Bragg curve influences DNA damage complexity and survival in head and neck cancer cells
Source: Clin Transl Radiat Oncol. 2025 Jan 3;51:100908. doi: 10.1016/j.ctro.2024.100908 (PMC11772976; doi:10.1016/j.ctro.2024.100908)
Supplement: Supplementary Data 1 [file mmc1.docx]

**Supplementary table 1:** Geant4-DNA parameters of the molecularDNA example used in the FaDu cell geometry.

| **Parameter “molecularDNA” example** | **Value** |
| --- | --- |
| $R_{dir} (Å)$ | 3.5 |
| $E_{min}^{break} \left( eV \right)$ | 5 |
| $E_{max}^{break} \left( eV \right)$ | 37.5 |
| $P_{OH}^{break} \left( eV \right)$ | 0.405 |
| $T_{chem} (ns)$ | 5 |
| $d_{kill}^{chem} (nm)$ | 9 |
